# Open-source, customizable phantom for low-field magnetic resonance imaging

**DOI:** 10.1007/s10334-025-01270-2

**Published:** 2025-06-25

**Authors:** Kalina V. Jordanova, Stephen E. Russek, Kathryn E. Keenan

**Affiliations:** Physical Measurement Laboratory, National Institute of Standards and Technology, 325 Broadway, Boulder, CO 80305 USA

**Keywords:** Phantoms, Imaging, Reproducibility of results, Magnetic resonance imaging

## Abstract

**Objective:**

This study aimed to describe important criteria for phantom design, while designing an open-source phantom that uses accessible materials and fabrication processes, and that can be easily reproduced and modified by others in the MRI research community.

**Materials and methods:**

We enumerate considerations related to designing a phantom based on literature and previous experience. We design and use an open-source phantom on a low-field MRI system. The phantom was 3D printed and assembled, and the imaged samples were made from commonly available materials. T1-weighted and T2-weighted axial and coronal images were acquired at 64 mT, and signal-to-noise ratio (SNR), contrast-to-noise ratio (CNR), and geometric distortion along one dimension were assessed for each image.

**Results:**

Two iterations of the phantom design were made to improve the construction materials and overall form factor for imaging. T1-weighted and T2-weighted images showed contrast between samples and background. T2-weighted images had an 8-10× increase in SNR and CNR compared to T1-weighted images. Geometric distortion measurements were within one-pixel spacing for all scans.

**Discussion:**

An open-source phantom was created to assess MRI scans at low-field. Future users may modify the phantom to suit their needs. User-designed inserts can be added, allowing for validation of many MRI-related measurements.

## Introduction

An imaging phantom is an object that can be evaluated, analyzed, or manipulated to study the performance of an imaging system [1]. When the phantom properties are well characterized, it can be a reference or calibration object for imaging. Phantoms can be composed of many different materials and are often designed to evaluate a specific imaging need.

MRI systems vary in field strength, machine size, coil form factor, etc. Additionally, MRI systems are used for a variety of imaging applications. Due to the large variation in MRI machines and imaging applications, many phantoms have been developed that target specific applications and are used for only a subset of MRI systems. For example, phantoms exist with structural elements to evaluate instrumentation [2], for structural brain imaging applications [3], with proton density fat fraction sensitive materials [4], for breast imaging [5], for diffusion measurements [6], for diffusion tensor imaging [7], for brain volume quantification [8], and for flow imaging applications [9]. Thus, similarly to the MRI systems and applications themselves, MR phantoms vary widely in structure and utility.

It can be difficult for research sites and institutions to purchase and maintain the many phantoms required to meet all system and imaging needs. Many commercially available phantoms are expensive, resulting in less frequent use than recommended [10], despite studies showing the utility in using phantoms to evaluate MR systems [11] and for quantitative methods [12]. The search for phantoms that are affordable, reproducible, and easy to assemble is ongoing, and recently researchers have even proposed using kiwi fruit as viable phantoms for prostate imaging [13]. There continues to be a need for low-cost, highly reproducible phantom designs for a plethora of MRI applications.

Open-source MRI hardware and tools can increase the technology’s affordability and accessibility [14–16]. Open-source phantom designs provide the ability to accurately evaluate MR systems and are good candidates for improving MR accessibility. Recent open-source designs include a pneumatic phantom to simulate human respiration using balloons [17], a preclinical phantom with a liquid-filled volume holding five micro-centrifuge tubes [18], a geometric distortion phantom [19], and an anthropomorphic brain phantom [20]. There is still a need for more general open-source designs that can be easily modified and adapted, as well as a comprehensive description of the considerations for designing open-source phantoms.

The first aim of this study is to describe the important criteria to be considered in phantom design. We highlight common mistakes that we and others have made in prototyping phantoms. The second study aim is to design an open-source phantom for low-field systems that uses accessible materials and design processes for its fabrication and that can be easily reproduced and modified by others in the MRI community. Additional goals for this phantom are to produce images with contrast using T1-weighted and T2-weighted scans, and to have the ability to assess geometric distortion along one dimension, signal-to-noise ratio (SNR), and contrast-to-noise ratio (CNR).

## Materials and methods

### Design considerations

We list the following considerations in designing a phantom, gathered from literature and from experience designing phantoms in our research group.

#### Phantom measurands

There are many measurands (measurement targets) for MRI applications and systems. They can be loosely categorized into system-related targets that assess how well an MRI system is functioning, and subject-related targets that measure properties of the scan subject. Examples of system-related targets include radiofrequency field uniformity, gradient amplitude, gradient linearity, geometric distortion, spatial resolution, slice thickness, slice position, image contrast, SNR, coil loading, and temperature. Examples of subject-related targets include relaxation (T1, T2, T1/T2, T2*, T1ρ), diffusion, fat fraction, fat suppression, elastography, proton density, magnetization transfer, susceptibility, chemical shift, flow, musculoskeletal imaging, conductivity, permittivity, and multimodal imaging signals (MR-PET, radiomics). There is overlap between system-related and subject-related targets: if the system is not functioning correctly, then the subject-related measurements will be affected.

#### Measurand materials

Phantom materials should be physiologically relevant for the target application(s). Ensuring that materials are representative of target tissues is vital to accurately evaluate scan performance for subject-related measurement targets. Even for many system-related targets, physiologically representative materials are necessary for assessing system performance for clinical use. Additionally, the properties of the phantom materials should cover the entire measurement range expected for the application. Finally, the materials should have a sufficient measurement resolution so that the results are useful for evaluating performance.

Beyond physiological relevance, phantom materials should be stable, and their properties should not degrade over their lifetime of use. Finally, phantom materials should not be toxic or harmful to the patient or operator. They should be affordable and easy to manufacture, which are both important for open-source designs.

A variety of materials have been used in MRI phantoms, each with its own properties and purpose. Some common materials are given in Table 1.

**Table 1:** Example materials used for MRI phantoms, categorized by their general applications

Material category	Example materials
Commonly used	Deionized water
Gelling agents	Agarose [41, 42], Carrageenan [43], Carbomer-980 and Carbopol-974P [44], Polyvinylpyrrolidone (PVP) [45]
Stabilizers	NaN_3_ [43], EDTA [46], Listerine [47]
Relaxation modifiers	Manganese [41], Nickel [48, 49], Gadolinium [26], Copper [42], gelling agents
Conductivity modifiers	NaCl [43, 50]
Permittivity modifiers	Denatured ethanol [50], PVP [51], sugar [52]
Chemical shift agents	Mineral oils [53, 54], Grapeseed oil [5], Peanut oil [55]
Flow and elasticity modifiers	Polyvinyl alcohol (PVA) [56–58]
Diffusion modifiers	Polypropylene yarns [59], PVP [60], Sucrose [61], Polyethylene glycol (PEG) [62], Acrylic fine particles [63]
Geometry modifiers	3d printable MR visible materials [64], Acrylic plates [65]

#### Physical properties

The physical design of a phantom includes the size, shape, and material. Many phantoms consist of multiple components. The arrangement of these components is also a design consideration. Finally, there are physical design choices that can improve phantom usability and safety.

##### Size and shape

A necessary requirement for any phantom is that it is small enough to fit into the imaging coil. The phantom should also load the coil similarly to how the human body would, so that the impedance matching circuitry and pre-scan functions can operate and be tested under similar conditions to human scanning. The electrical permittivity and conductivity of the phantom materials will also affect coil loading.

The phantom’s shape should minimize air interface susceptibility artifacts. These artifacts are often minimized by creating a phantom that consists of a liquid-filled container, thereby reducing the surface area that interfaces with air. Other design choices for phantom shape include whether it is symmetric or asymmetric, and whether it is designed to mimic the anatomy of a specific organ or tissue.

##### Shell material

The material of the phantom shell or casing is distinct from the measurand materials in that the primary task is to encase the measurand materials. An encasing material should not produce artifacts in the MR image. As with the measurand materials, the shell material can be chosen to target MR properties. For example, the magnetic susceptibility of the shell can be selected to mimic in vivo conditions [21, 22]. The outside of the phantom can be made flexible by using a material that is pliable, such as silicone or rubber [5], allowing the phantom to conform to the coil shape and to mimic pliable anatomy. Additive manufacturing can be used to create the phantom shell.

##### Phantom components

MRI phantoms are generally made up of components, some targeting desired measurands and some required for usability. For tissue mimicking samples, the sample size depends on the available space and desired image resolution. Samples can be arranged in a grid to offer the ability to do geometric assessment. Tissue mimicking samples are often placed in a fillable volume. When designing a fillable phantom, two fill ports, large enough for easy filling and releasing air, should be included.

Positioning aids for orienting the phantom in the scanner include 3D-printed serial numbers or text visible in the MR image, and levels or crosshairs to orient the phantom when it is placed in the scanner. It is desirable to have some MR visible asymmetry for image orientation, as well as features that extend through the sagittal, axial, and coronal imaging planes to help with image registration.

For temperature regulation and monitoring, a thermometer can be placed through a fill port, or an MRI-readable thermometer can be included within the phantom [23]. Often ice water or flow jackets are used to fix the temperature, but these techniques make imaging more complex.

##### Physical design for usability

To make the phantom easy to handle, it should be as light as possible and easy to move. If the phantom is spherical, a holder can help with scanner positioning. A holder can also help ensure the phantom is always in the same position in the scanner, which will improve measurement repeatability. The phantom should be easy to assemble, and the bolts that hold it together should be easy to tighten and loosen. Phantoms with modular designs may provide greater usage flexibility.

##### Physical design for safety

Ferromagnetic materials should be avoided in the phantom materials as well as in fasteners used in the phantom. Some materials may have unexpected or unknown ferrous compositions (e.g., epoxies or pigments). Safety data sheets or a description of safe consumption-approved components should be provided with the phantom. A short safety response plan should be provided in case the phantom is damaged and leaks, even if it is composed of safe nontoxic materials. Pressure release or expansion ports may be required if the phantom is designed to operate over a wide range of temperatures to ensure the phantom does not crack or pose an over-pressure safety hazard.

#### Software and scan protocols

Sequences used in the phantom scan protocol should be the same as in in vivo imaging for subject-related measurands. Longer “gold standard” sequences may be appropriate for system-related measurands. The range of acceptable scan parameters should be identified for each sequence. The positioning of the phantom in the scanner should be pre-defined in the design process.

Ideally, data analysis tools would be provided so that the analysis is standardized across users and sites [8]. Some key aspects of a data analysis toolbox include how to define regions of interest (ROI), how to calculate statistics such as accuracy, variation, and bias, and how to determine confidence in measured values. A toolbox to measure image characteristics between different hardware or software versions (e.g., image scaling calculations) would be useful. A toolbox could also record variations in temperature, field strength, or other quantities that could affect measurements.

Finally, publishing numerical three-dimensional descriptions (e.g., computer-aided design (CAD) files, recommended ROI locations, property maps) of open-source phantoms allow for experimental simulations using the phantom. These numerical descriptions constitute a corresponding digital phantom. Creating a public database or forum where users can share information on protocols and measurements can further aid in widespread adoptability of phantom use.

### Open-source phantom design

#### Phantom measurands

The goal for the phantom design was to create an accessible phantom using off-the-shelf materials that exhibit contrast in T1-weighted and T2-weighted images, as T1-weighted and T2-weighted scans are very common and can be found on most MRI systems. A second aim for the phantom design is the ability to target system-related measurands, in this case geometric distortion along one axis, SNR, and CNR.

#### Measurand materials

For open-source phantoms, it is important that the materials are accessible and affordable, in addition to the requirement that they are sensitive to the measurands. This phantom design uses deionized water, olive oil (Whole Foods, Austin, TX, USA), and Chelated Zinc plus Copper mineral supplements (Whole Foods, Austin, TX, USA), which were bought at a local store. Oil is a common material used as an approximate fat mimic, whereas copper is a T1 and T2 modifier. Copper supplements were dissolved in deionized water in 15 ml falcon tubes (Fisher Scientific, Hampton, NH, USA), at varying concentrations. These solutions are straightforward to manufacture, as opposed to more process-involved materials (e.g., materials with gelling agents or PVP). Three tubes were filled with one, two, or three copper supplement capsules, respectively, resulting in sample concentrations: 15 mg Zn and 2 mg Cu; 30 mg Zn and 4 mg Cu; and 45 mg Zn and 6 mg Cu.

#### Physical properties

The phantom was designed to be a fillable cylinder with an 18 cm outer diameter and 14.8 cm inner diameter. The 18 cm diameter of the cylinder was chosen to be compatible with the OSI^2^ ONE [24] open-source MRI system, which has a 20-cm-inner diameter RF coil. Two tops to the cylinder were designed: a flat top resulting in a phantom length of 16 cm, and a domed top resulting in a phantom length of 21.4 cm.

The phantom can hold up to 24 samples of target material (in this case, water, oil, or dissolved supplements). Two plates were designed to hold samples, each of which can slot into the phantom volume and has holes that sample tubes press fit into. The first plate is a rectangular plate oriented along the length of the cylinder, and it can hold 15 of the 15 ml Falcon tubes and 9 smaller 5 ml cryogenic tubes (Wheaton Science Products, Millville, New Jersey, USA). The second plate is a circular plate oriented coaxially with the cylinder, and it can hold 18 of the 15 ml Falcon tubes and 6 of the 5 ml cryogenic tubes.

### Phantom fabrication

**Fig. 1:**
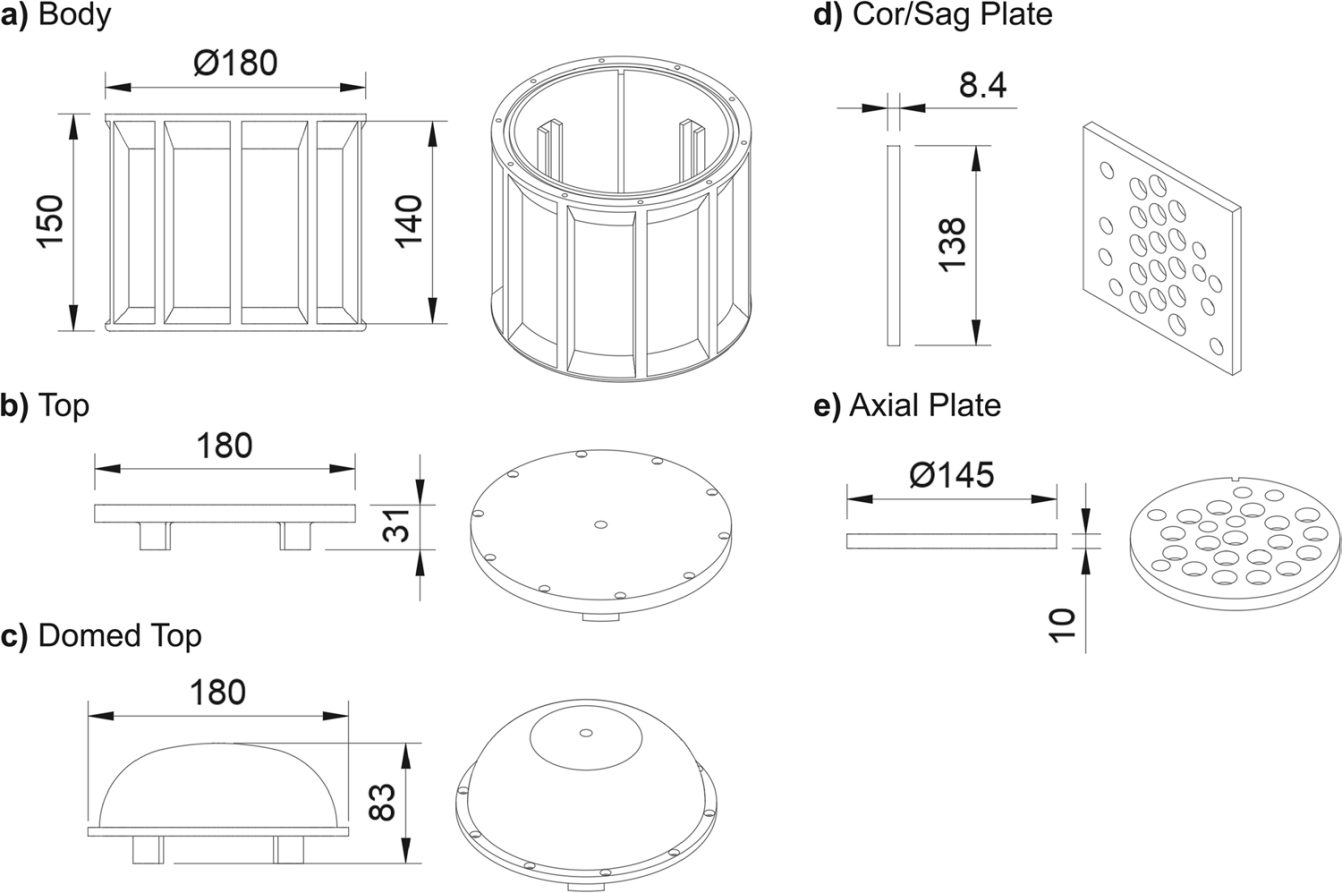
CAD model of the open-source phantom where dimensions in millimeters are indicated by double-sided arrows. **a** The body of the phantom, which can be filled with a liquid. **b** The top of the phantom, used as a liquid-tight seal. **c** A domed version of the phantom top. **d** A plate that can be inserted into the phantom to hold sample tubes. This plate is referred to as the Cor/Sag plate because it is often oriented in the MR scanner such that coronal or sagittal images will show cross sections of tubes held in the plate. **e** A plate that can be inserted into the phantom to hold sample tubes. This plate is referred to as the Axial plate because it is often oriented in the MR scanner such that axial images will show cross sections of tubes held in the plate

The phantom (Fig. 1) was designed using the CAD software Fusion 360 (Autodesk, San Francisco, CA, USA). STL and STEP files of the design have been made available. The outer shell of the phantom was printed on an Original Prusa 3D printer (Prusa Research, Prague, Czech Republic) using gray polylactic acid (PLA). A fused deposition 3D printer was used since they are common and inexpensive. Two test plates were printed on the Prusa printer using black PLA material. Duplicates of the test plates were printed on a Form 3 printer (Formlabs, Somerville, MA, USA) in a stereolithography (SLA) material. After printing and before filling, the fill port and screw holes were drilled out using appropriate drill bits for each hole and tapped with a threading tap.

To seal the phantom for liquid-tight performance, the inner lining of the outer shell was coated with two coats of white Plasti Dip (Plasti Dip International, Blaine, MN, USA). Alternatively, the shell can be printed using a photocured resin (SLA or digital light processing (DLP)), that is watertight, without the need for extra sealing. A 4-mm-wide, 155-mm-inner diameter oil-resistant Buna-N o-ring (part number 1302N303, McMaster-Carr, Elmhurst, IL, USA) was placed between the phantom body and top. This o-ring sizing affected the dimension of the phantom’s inner chamber, resulting in a phantom inner diameter of 14.8 cm. The top was bolted into place using ten 14-mm-long M4 × 0.70 mm thread nylon plastic socket head screws (part number 93640A146, McMaster-Carr, Elmhurst, IL, USA). Although nylon is typically avoided inside phantoms because it absorbs water and deforms, it can be used outside of the water volume. Finally, to plug the phantom after filling, a 1/8 NPT pipe plug (part number 45505K195, McMaster-Carr, Elmhurst, IL, USA) was used.

The phantom was assembled by press fitting the sample tubes into the selected tube-holder plate, placing the plate and tubes into the phantom, and filling the phantom with deionized water to the top of the cylinder. The top was bolted on, and the phantom was filled with more water through the fill port until it overflowed. Finally, the fill port was plugged.

### Data acquisition

Axial and coronal T1-weighted and T2-weighted images of the phantom were acquired using a 64 mT Hyperfine Swoop (version 1.8, Hyperfine, Guilford, CT, USA). T1-weighted images were acquired using a multi-slice inversion-recovery spin-echo sequence with a repetition time of 1.5 s, inversion time of 0.3 s, echo time of 0.006 s, 24 signal averages, in-plane resolution of 1.6 mm × 1.6 mm, slice thickness of 5 mm, and fields of view of 22 cm (anterior/posterior) or 18 cm (superior/inferior and left/right). T2-weighted images were acquired using a three-dimensional fast spin-echo sequence with a repetition time of 2 s and an echo time 0.231 s (coronal imaging) and 0.203 s (axial imaging), and 80 signal averages. The in-plane resolution was 1.5 mm × 1.5 mm, and the slice thickness was 5 mm, with fields of view of 22 cm (anterior/posterior) or 18 cm (superior/inferior and left/right). The acquisition times for the T1-weighted images were 5:31 (coronal imaging) and 5:37 (axial imaging). For the T2-weighted images, the acquisition times were 5:25 (coronal imaging) and 5:55 (axial imaging).

To evaluate geometric distortion along one axis, the distance between sample vial centers was calculated for a central slice of the scans: T1-weighted coronal scan using the flat top; T2-weighted coronal scan using the flat top; T1-weighted coronal scan using the domed top; T2-weighted coronal scan using the domed top. The center of each vial was found by identifying each vial’s region of interest (ROI) through an automated process of first thresholding each image so that the edges of the vials were visible, and then finding representative circles for each vial edge. Once all ROIs were determined for an image, the inter-vial distance was calculated between circle centers for consecutive vials. Using all inter-vial distances, the average inter-vial distance was calculated for each image. To interpret the inter-vial distance metric, note that this phantom did not include external or internal level indicators, so it could have been placed at an angle to the imaging volume. Thus, the average inter-vial distance cannot be directly compared to the absolute inter-vial distance of the phantom. Instead, comparisons of the average inter-vial distances between scans with the phantom placement unchanged can be made to determine whether there is relative distortion between scan types. Here, average distance comparisons can be made between the T1-weighted and T2-weighted scans for either the flat top or domed top images.

To evaluate the SNR and CNR, two noise regions of 10 × 10 pixels near the edge of the images were selected, as well as one signal region of 5 × 5 pixels. For T1-weighted images the signal region was placed within the vial of highest signal intensity (oil). For T2-weighted images the signal region was placed in a centrally located portion of the background water fill, as the water signal had the highest intensity in the T2-weighted images. SNR was calculated as the ratio of the mean signal value to the standard deviation of the noise pixel values. CNR was calculated as the ratio of the difference of the mean signal value and the mean noise value to the standard deviation of the noise pixel values.

## Results

### Open-source phantom design

Figure 1 shows CAD images of the initial open-source phantom design. The full design is provided (linked in Data availability statement). Figure 1a shows the phantom body, designed to be a fillable cylinder to minimize air interface artifacts. Figure 1b shows the flat version of the phantom top, and Fig. 1c shows the domed version of the phantom top. The two plates that hold sample tubes are shown in Fig. 1d–e. The rectangular plate that aligns lengthwise with the cylinder is called the “Cor/Sag” plate, because coronal or sagittal images will show cross sections of the tubes held in the plate. The plate that aligns coaxially with the cylinder is called the “Axial” plate, because axial images will show cross sections of the tubes held in the plate. The Axial plate sits atop the interior ridges of the body and is held in place using pressure from the protruding flange on the phantom top.

**Fig. 2:**
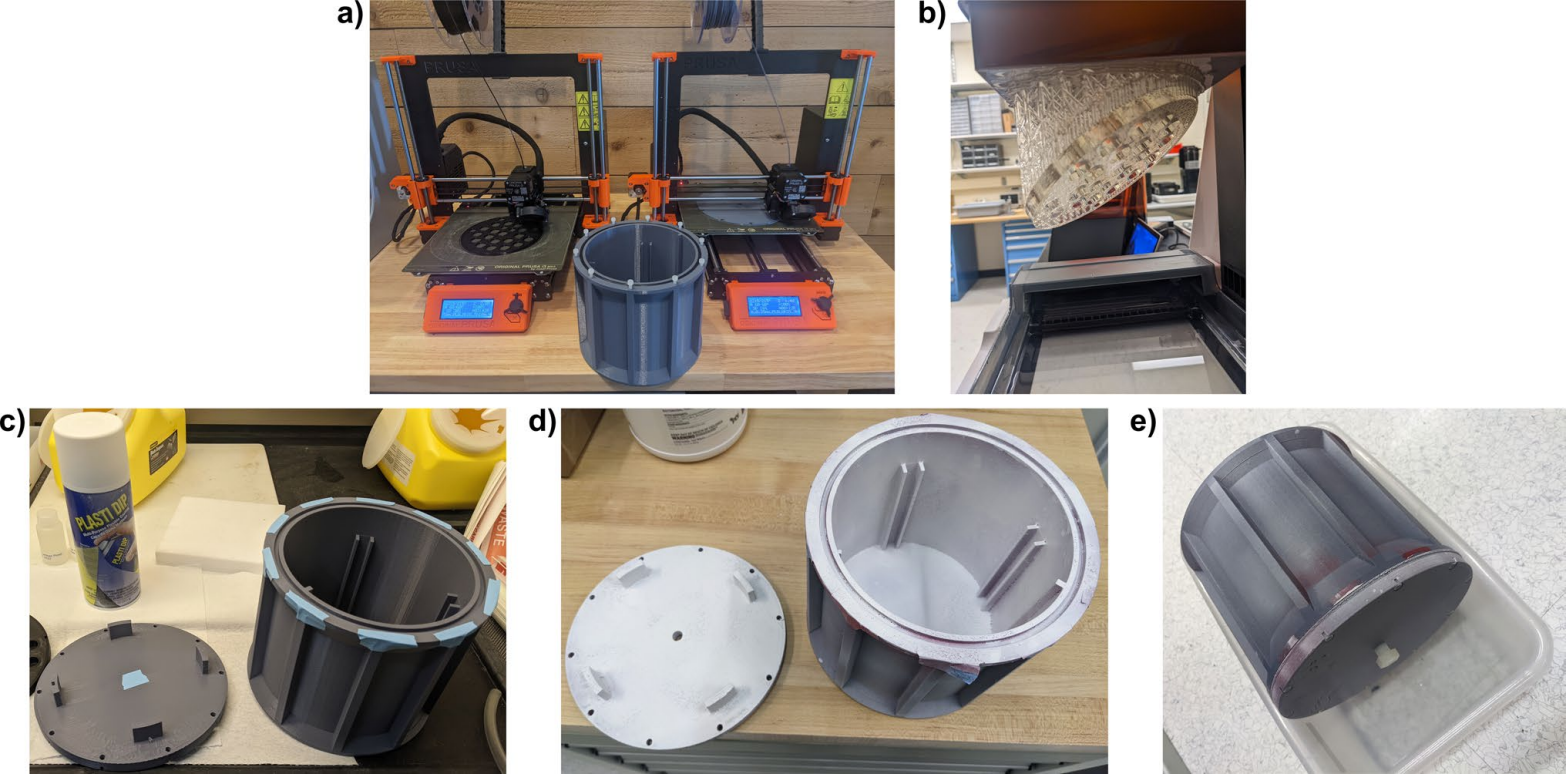
Phantom manufacturing process. **a** The body and top of the phantom were constructed on an Original Prusa 3D printer. The plates were printed on the Prusa, as well as on a Formlabs Form 3 printer. **b** The sample holding plates were printed on a Formlabs Form 3 printer. **c** The top and bottom of the printed phantom. **d** The interior of the top and bottom of the phantom were sealed with Plasti Dip to prevent leakage. **e** Leakage test of the phantom filled with water

Figure 2 shows the phantom manufacturing process. Insertable plates were printed on a Prusa and a Formlabs Form printer. After printing, the phantom housing was sealed using Plasti Dip so that it would not leak when filled. An image of a fill-leak test is shown in Fig. 2e.

### Phantom performance

**Fig. 3:**
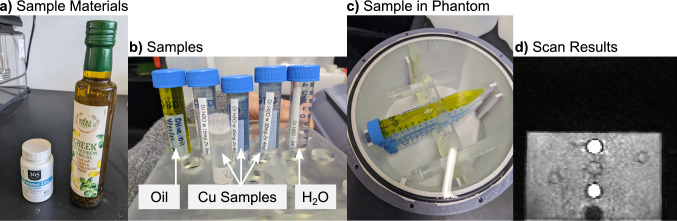
**a** Sample materials included deionized water, olive oil, and dissolved supplements that can be found in a store. **b** Sample tubes filled with oil, different concentrations of copper from the dissolved supplements, and deionized water. **c** Sample tubes placed into the phantom using the Cor/Sag plate. The phantom is filled with deionized water. **d** Coronal T1-weighted MRI scan that shows contrast between the oil, copper samples, and water fill

All materials used for the samples (Fig. 3a) can be found in stores. Figure 3d shows a T1-weighted image of the samples. Oil is a common material used as an approximate fat mimic and appears contrasted to water in the T1-weighted image. Similarly, because copper is a T1 and T2 modifier, the three copper samples have different image contrast from each other and from oil and water. The linear placement of the sample tubes in Fig. 3d could be used to assess geometric distortion in the image.

**Fig. 4:**
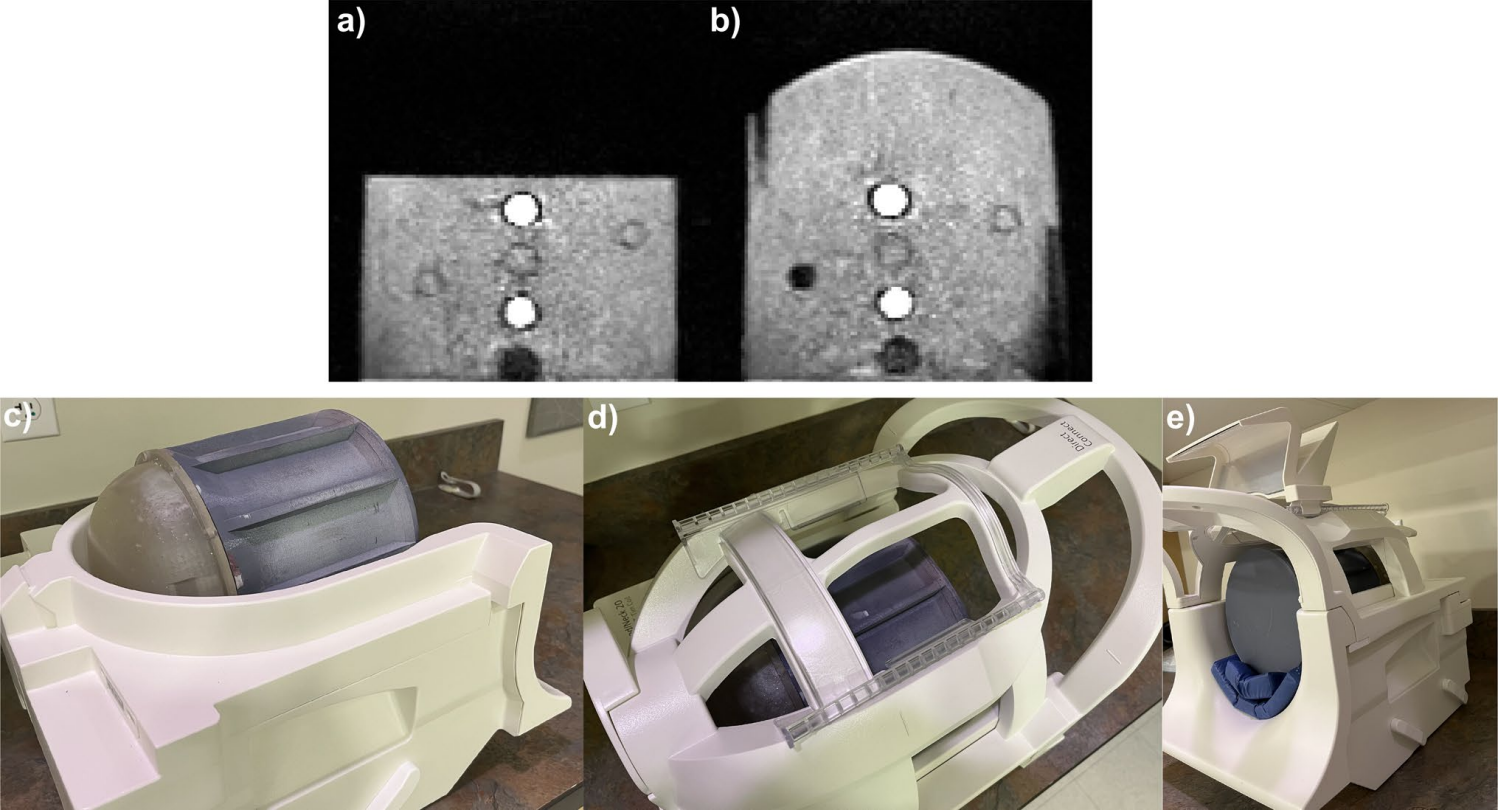
**a** T1-weighted coronal image using the flat top at 64 mT. **b** T1-weighted coronal image using the domed top at 64 mT. **c**–**d** Photos showing the fit of the phantom with domed top in a 3 T, 20-channel head coil

The image in Fig. 3d was acquired using the flat top. Additionally, a modified top with a dome shape was designed to better fill the coil. Figure 4 shows the comparison of the T1-weighted images using the flat and domed tops. Figure 4c–d show that the phantom with the domed top fits into a Siemens 20-channel head/neck coil (Siemens Healthcare, Erlangen, Germany).

**Fig. 5:**
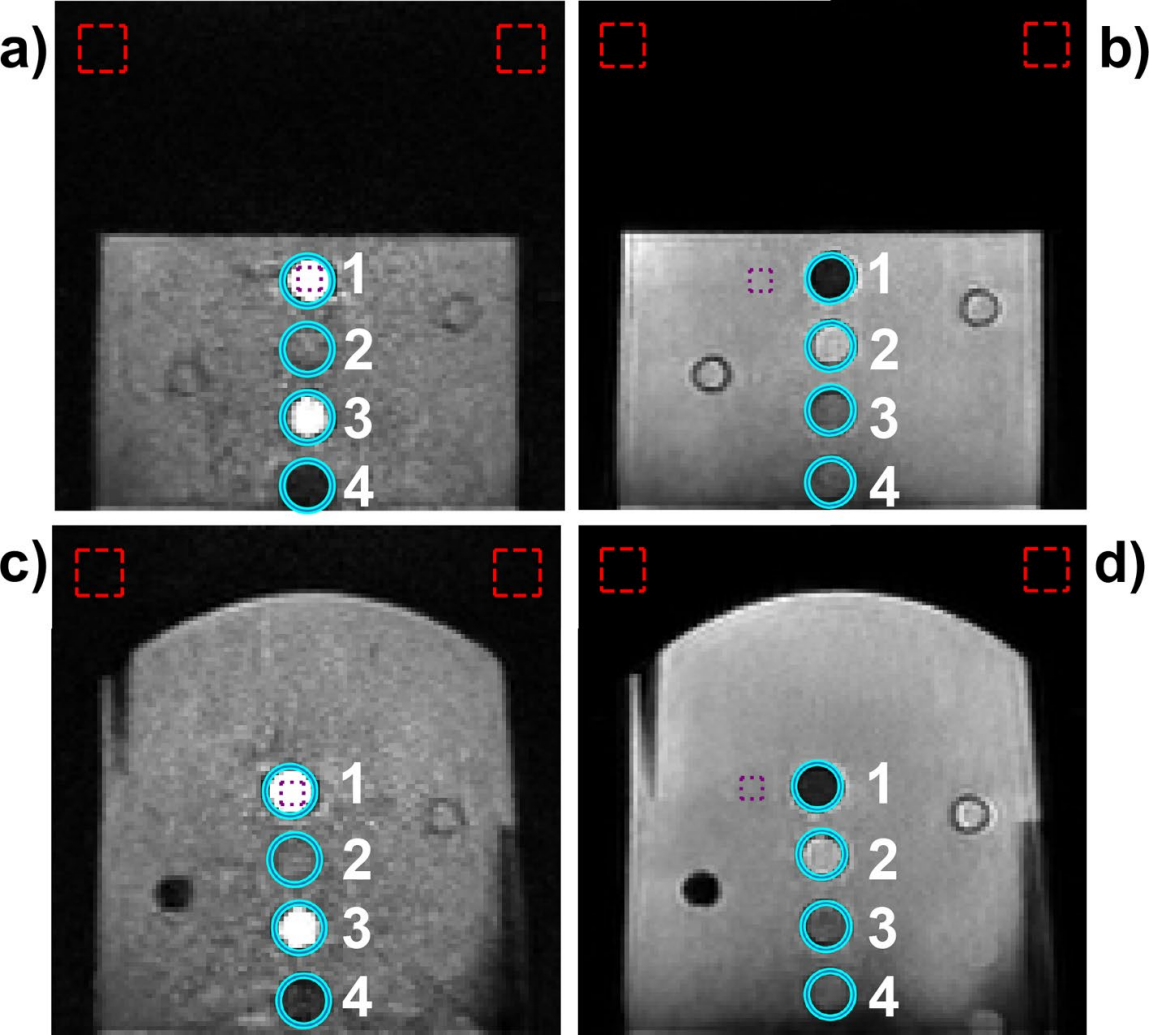
64 mT T1-weighted (**a**, **c**) and T2-weighted (**b**, **d**) images for flat top (**a**, **b**) and domed top (**c**, **d**) coronal plate acquisitions. Geometric distortion was calculated for each image using the distance between the center of consecutive ROIs (cyan circles, numbers indicate consecutive ROIs). Rectangles mark two noise regions (red) and one signal region (purple) used for SNR and CNR calculations

The phantom was used to evaluate geometric distortion, SNR, and CNR for each coronal T1-weighted and T2-weighted image acquired at 64 mT. Figure 5 shows a central slice for two coronal plate images (T1-weighted and T2-weighted) using the flat and domed tops, along with circular ROIs for each sample vial. Consecutive inter-vial (inter-ROI) distances were calculated and are given in Table 2. It is noted that distances cannot be compared between the flat and domed top images, as those involved moving the phantom, and the phantom positioning was not repeatable. Instead, comparisons between T1-weighted and T2-weighted distances can be made within the flat or domed top images, as those were taken consecutively without phantom movement. For both the flat and domed top images, the difference between average inter-vial distances (0 mm for the flat top, 0.1 mm for the domed top) were well within the image pixel spacing (1.6 mm for T1-weighted, 1.5 mm for T2-weighted), indicating zero or very little geometric distortion in the acquired images for regions near the sample vials. The SNR and CNR are comparable for the flat and domed top versions (Table 2). The SNR and CNR are each 8–10 × larger for the T2-weighted images than for the T1-weighted images.

**Table 2:** Distances calculated between consecutive vial ROIs for T1-weighted and T2-weighted coronal scans with either the flat top or domed top. Average distances for each scan are calculated. SNR and CNR for each image are also given

	Distance (mm)				SNR	CNR
	Vial 1 to vial 2	Vial 2 to vial 3	Vial 3 to vial 4	Average		
T1-weighted, flat top	24.0	24.0	24.0	24.0	332	328
T2-weighted, flat top	24.0	22.5	25.5	24.0	2487	2481
T1-weighted, domed top	24.1	24.1	25.6	24.6	257	254
T2-weighted, domed top	24.0	25.5	24.0	24.5	2505	2498

## Discussion

We designed and tested an open-source phantom for low-field imaging that is fillable and can hold user-selected samples that are oriented in either an axial, coronal, or sagittal configuration. The phantom is a cylinder with either a flat or domed top. We demonstrated the phantom’s ability to show MRI contrast between materials easily sourced from stores. One part of the utility of an open-source design is that this design can be modified by users to be scaled to fit different measurement systems.

### Design considerations

In this study we presented considerations for designing phantoms and reference objects, gathered from literature and from our group’s experience in phantom design. We hope these criteria will aid in increasing open-source phantom design availability.

Identifying the measurands for a phantom is a critical first step in phantom design, because they determine many subsequent design criteria. For example, the phantom materials and form factor are impacted by the measurands. Although phantom materials should have physiological relevance, it can be difficult to know the range of physiological parameters for a given measurand, and previous literature may need to be consulted [25–29]. A complication in achieving physiological relevance is that subject-related measurands can change even in the low-field strength regime [25–27]. Finally, sample materials should be repeated in the phantom so that measurement variability can be assessed at multiple positions within the scanner.

In general, material stability is important but can be difficult to achieve for long-term studies. Often, a tradeoff exists between material stability and how biomimetic a material is. It is also important to choose an affordable and easy to manufacture shell material. Additive manufacturing is a good option for creating phantom shells, because it is a relatively quick and easy way to create phantom designs and to share them broadly. This makes additive manufacturing technology particularly attractive for open-source designs. It is a heavily researched topic in MRI, with recent developments in MR visible 3D-printed materials [30]. Please note, in liquid-filled designs, it is important to avoid using nylon, as it will absorb water and deform [31], which is particularly troublesome for use as a geometric reference object.

MR systems’ sizes and shapes vary widely; therefore, the phantom size and shape should be chosen for compatibility with the target system and application. It is noted that minimizing air interface area is especially important for phantoms used in fields > 1.5 T, where air interface artifacts can be significant [32]. The field strength can also be considered when determining the phantom size and shape. Depending on the phantom size, shape, and orientation relative to the main field, the field perturbations can vary. Spheres and long cylinders are often selected due to ease in simulating their effect on field perturbations [21]. Acquiring calculated and measured field maps can help in understanding the homogeneity of the design.

Usability is important for any phantom. For open-source designs, the usability can determine how widely the design is adopted. There are a few factors in the phantom’s physical design that improve usability. Designing modular phantoms can improve usability. For example, a recent study designed a modular phantom with different LEGO-compatible components that could be combined to build a phantom of any shape, size, and composition [33]. Components spanned a variety of targets, including a resolution grid and fillable components that could be used to measure T1 and T2 of a known sample. Designing for phantom usability includes considering ways to make it easier for the user to evaluate their system performance using the phantom. This may include defining scan protocols that target the measurand, providing data analysis tools, and providing a digital version of the phantom.

Temperature measurement and control is critical for many measurands including relaxation times and diffusivity. Bore temperatures can vary (18 °C–24 °C), and thermal equilibration times can be long.

Finally, it is important to consider safety when designing phantoms. A good safety measure is to test all phantom components with a magnetic wand before use.

### Open-source phantom design, lessons learned

The first version of the design included a flat top for the cylinder. To extend the phantom height, a domed top was designed. An added feature of the domed top is that it acts as an air bubble trap since bubbles will move toward the center of the dome while filling. The dome further allows better fits in standard head coils. The base of the phantom could also be domed to make the phantom symmetrically domed on both ends; however, filling the phantom with a domed base may be challenging and may require a support structure.

Another issue presented itself when sealing the phantom using Plasti Dip. After sealing, the phantom interior was slightly thicker due to the layers of Plasti Dip, and the plates that held the samples were difficult to fit into their respective slots. Thus, future designs should leave extra tolerance for nesting parts if adding a sealant after printing or using SLA or DLP printing instead of fused deposition.

The current phantom design has only one fill port. This makes filling the phantom difficult, as there is no air release port. A future design should add another port for air release. Additionally, changing the sample tubes once the phantom is filled can be time consuming and messy. An improved design would have the phantom fill contained in a separate compartment from the sample vials. This can be done by creating a container to hold the phantom fill with holes that vials are inserted into (see Fig. 2 in [34] for example). To reduce air bubbles, the phantom can be rinsed with isopropyl alcohol prior to filling. Slightly shaking the phantom after filling can also help dislodge air bubbles, which can then be aggregated and replaced with water.

**Fig. 6:**
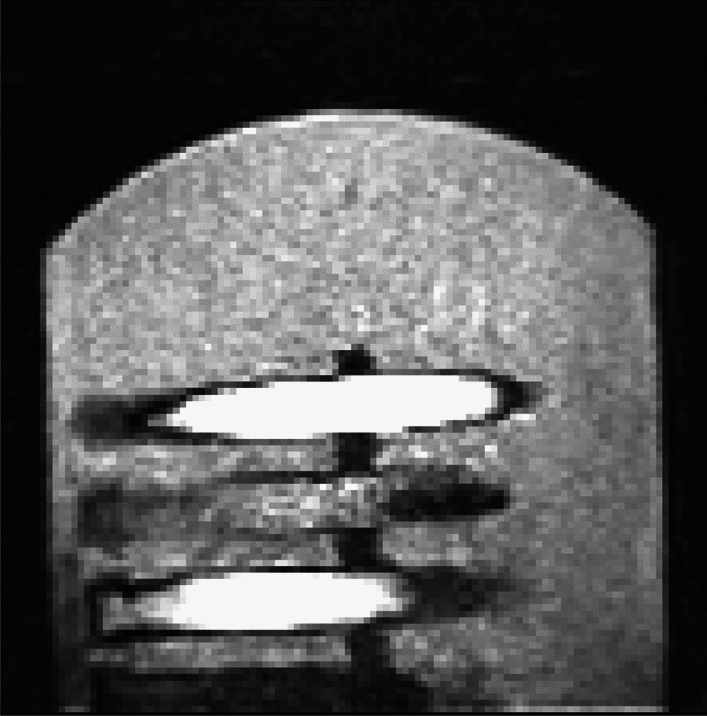
64 mT T1-weighted Coronal image using the domed top with the sample tubes not aligned

A few revisions of the phantom were already identified in the initial fabrication. The design did not include visual exterior markings to aid in scanner orientation, so the initial placement of the domed top version was placed into the scanner with the cross section of the samples oriented sagittally (Fig. 6) rather than coronally, as desired. Previous studies have shown imaging artifacts from black ink [35]. In this study, the interior plates were originally printed in black, and then reprinted in a nonblack material.

A final design improvement is to use sample materials with known relaxation times. This can be achieved by mixing materials based on published recipes, or when using materials from the store. In both cases, it is necessary to obtain a reference measurement of material relaxation properties using methods such as inversion-recovery for T1 and single-echo spin-echo for T2.

### Phantom performance

To evaluate phantom performance, we used materials found in stores to create contrast in T1-weighted and T2-weighted images at 64 mT. Both T1-weighted and T2-weighted scans showed image contrast between sample tubes, as well as between the tubes and the background water fill. In this study, oil and a zinc-copper supplement dissolved in water were used to create relaxation-based contrast. These materials have the advantage that they are nontoxic and do not need hazardous waste disposal. The sample materials were selected with short-term use in mind, such that they could be refabricated on an as-needed basis. If the intended use of these materials were to be long-term, then their stability would need to be evaluated. The phantom is designed so that future studies can use different contrast materials of choice. It is straightforward to swap the sample materials for different electromagnetically and physiologically correct tissue mimics. For example, materials that exhibit specific T1 or T2 relaxation properties could be used in the phantom for quantitative applications [36].

Coronal scans were acquired using T1-weighted and T2-weighted protocols with both the flat and domed tops. Geometric distortion was calculated for each scan by determining average inter-vial distance for four sample vials. While there was variation in individual inter-vial distances for each scan type, the variation was less than one-pixel spacing and could be accounted for in a one-pixel inaccuracy of the ROI center identification process. Thus, the average inter-vial distance is a better representation of distortion. For both the flat and domed top scans, the T1-weighted and T2-weighted scans had average inter-vial distances within 1% of each other, indicating zero or very little geometric distortion between those two scan types. The domed top has merit as a design, because it is more representative of the human head and better fills the coil. The evaluation here is rudimentary and served as a proof-of-concept rather than a rigorous geometric assessment. As a next step, tubes can be incorporated into the grid in two dimensions rather than one. Further, a more robust method would incorporate 3D grids into the modular phantom design. Additionally, a basic analysis of SNR and CNR was conducted for each scan result, contributing to the growing body of work to measure SNR and CNR for low-field systems [37–40]. The results were comparable for domed or flat top, while the T2-weighted scans had a significant increase in SNR. Although many of the scan parameters for this low-field system are proprietary and therefore obscured, the SNR increase for the T2-weighted images is expected in part due to the increased number of averages for that acquisition (80, versus 24 for T1-weighted).

## Conclusion

We described the important criteria to be considered in phantom design and designed an open-source phantom that uses accessible materials and manufacturing processes. The phantom was manufactured using 3D-printed materials and easily sourced products were used to create samples for scanning. It is straightforward to swap the sample materials for a variety of samples that can serve as tissue mimics. The design presents a way to test out a diverse set of phantom components, allowing for additional modular insets, such as slice profile wedges, resolution insets, and geometric distortion insets to be made. The phantom was used to assess geometric distortion in one dimension, SNR, and CNR across four scan types using a low-field system. This phantom can be easily reproduced and modified by others in the MRI research community.

## Data Availability

The code that supports this study will be made openly available upon publication at this link: https://github.com/usnistgov/open-source-mri-phantom
